# Exploring health navigating design: momentary contentment in a cancer context

**DOI:** 10.1080/17482631.2017.1374809

**Published:** 2017-09-14

**Authors:** Ulrika Sandén, Lars Harrysson, Hans Thulesius, Fredrik Nilsson

**Affiliations:** ^a^ Department of Design Sciences, Lund University, Lund, Sweden; ^b^ School of Social work, Lund University, Lund, Sweden; ^c^ Department of Clinical Sciences, Lund University, Malmö, Sweden

**Keywords:** Safety, moment, cancer, patient, grounded theory, innovation, happiness, design

## Abstract

**Purpose:** The technocratic and medicalized model of healthcare is rarely optimal for patients. By connecting two different studies we explore the possibilities of increasing quality of life in cancer care.

**Methods**: The first study captures survival strategies in a historically isolated Arctic village in Norway resulting in *Momentary contentment theory*, which emerged from analysing four years of participant observation and interview data. The second study conceptualizes everyday life of cancer patients based on in-depth interviews with 19 cancer patients; this was conceptualized as *Navigating a new life situation*. Both studies used classic grounded theory methodology. The connection between the studies is based on a health design approach.

**Results**: We found a fit between cancer patients challenging life conditions and harsh everyday life in an Arctic village. Death, treatments and dependence have become natural parts of life where the importance of creating spaces-of-moments and a Sense of Safety is imminent to well-being. While the cancer patients are in a new life situation, the Arctic people show a natural ability to handle uncertainties.

**Conclusion**: By innovation theories connected to design thinking, *Momentary contentment theory* modified to fit cancer care would eventually be a way to improve cancer patients’ quality of life.

## Introduction

Innovation is defined as the creation of something new that provides value to a specific customer, patient or user (Christensen, Grossman, & Hwang, 2009; Nilsson & Lindström, 2010; West, 1990). At the same time “…*innovation in health care* is defined as those changes that support health care practitioners focus on the patient by helping health care professionals work smarter, faster, better and more cost effectively” (Thakur et al 2012, p. 564). In healthcare a dominant view on innovation is that it is gained from external and formal research programs that are transferred to practice as a final step (Herzlinger, 2006). In other words, the technocratic and medicalized model of healthcare is rarely optimal for patients, but rather internally focused, that is, do not include the patient in the innovation process.

*Momentary contentment theory* emerges from a context where accidents and deaths are part of life and where helping each other is a necessity (Sandén, Harrysson, & Thulesius, 2015; Sandén, Thulesius, & Harrysson, 2015). In theory it has a potential fit with cancer patients where death is often a possible outcome. In order to explore the potential practical use of the theory we have identified different strategies (see results section), which in a health context may work towards inclusion, safety and contentment. Momentary contentment theory is a classic grounded theory stemming from exploring safety and life choices within a small historically isolated Arctic Norwegian community. It shows mechanisms that have emerged from the historically rough living conditions dealing with the present moment, taking one incident at a time. This relative conception of time, however, stands in contrast to today’s busy society. When helpfulness is a priority it means that people are delayed because they meet someone who needs help. In a comparative study between the various regions in Norway, Vea (2009) discusses how the prioritizing of the moment in Northern villages creates a lack of macro innovative economic thinking.

In a cancer patient study, we found a fit in both context and needs with Momentary contentment theory. The cancer patients show a fragmented way of searching for momentary contentment strategies towards inclusion, safety and a happier moment. We call this process *Navigating a new life situation*. In modern society’s focus on economic growth it is hard to adopt a different way of viewing time and creating space in the moment. This may, however, be doable in order to find better ways of living with illness–adding a humanistic perspective to the technocratic medicalized model of healthcare.

**Figure 1.**
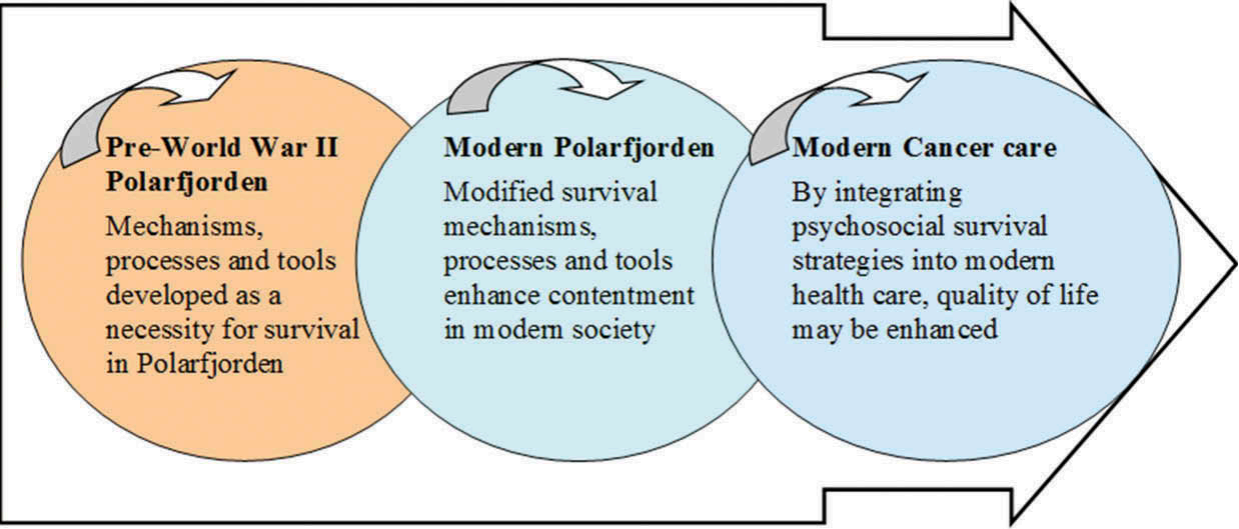
A “recycling” process of psychosocial survival strategies.

Innovation potentials emerge out of the expressed needs of users and patients. Ackoff (1999) discusses problems as being a system of problems rather than isolated phenomena. Thus, solutions need to consider the whole system–a total contrast to patient testimonies in our interviews, where fragmented healthcare was an obvious obstacle to health. In Momentary contentment theory, we find a possible innovative frame of reference based in a similar context of living as for cancer patients. Our aim was to connect Momentary contentment theory and data from a cancer patient study with an innovative discourse on how to help individuals with serious illness (Figure 1). In this article we present the results as a contrasting approach we chose to call *health navigation design* where patients and their needs are at the centre in evolving health practices.

## Methods

We used classic grounded theory (Holton & Walsh, 2017) to generate both studies resulting in *Momentary contentment theory* and *Navigating a new life situation*. When exploring the possibilities of connecting the two studies we chose to use design thinking and innovation theories as a means to be patient inclusive.

### Research design

This article is based on two empirical research projects using classic grounded theory methodology in data collection, sampling and analysis. We started with a longitudinal observational study of a partly isolated Arctic village, complemented with interviews (Sandén, Harrysson et al., 2015). We then did a second study focusing on needs of cancer patients based on interviews. Finally, we did a third analysis connecting the two studies. In order to address the social innovation dimensions and to go from theoretical reasoning to innovation suggestions, we have adopted a design thinking perspective. Design thinking “addresses the needs of the people who will consume a product or service and the infrastructure that enables it” (Brown & Wyatt, 2010, p. 32). It was apparent in our interviews with cancer patients that they struggled with navigating in a new and unfamiliar life situation. We chose to explore what we call *health navigation design*, that is, the design of individual patient’s relationship to and communication with relatives, with other patients, with healthcare personnel, with products and with other significant people in their lives.

### Data collection

#### Part one: “Momentary contentment”

*- Conceptualized data from four years (2010 to 2014) of interviews, talks and observations of everyday life surrounding an Arctic fjord, “Polarfjorden”*. In accordance with the classic grounded theory dictum “All is data” Glaser, 1998) all written notes from observations, diaries, formal and informal interviews were coded and compared in discussions between the authors. Theoretical memos were written in conjunction with the coding and comparisons. A total of six focus group interviews and eight individual unstructured as well as semi-structured interviews were conducted. Each interview lasted between 2 and 6 hours. In order for us to capture the informant’s views of their everyday lives they were asked to freely talk about their experiences. Some of the later interviews were semi-structured in order to catch a specific social aspect. We also analysed field notes from 15 targeted conversations and 50 informal, semi-structured conversations with both women and men from Polarfjorden and adjoining areas. All informants were shown the transcripts from their interview as well as of field notes from conversations and observations. They were given the possibility to suggest corrections due to misinterpretations. No informant suggested corrections.

### Part two: “Navigating a new life situation”

##### Interviews with cancer patients

Detailed fieldnotes were collected from six focus-group interviews, two individual interviews and a follow-up individual interview with one cancer patient in 2015 (Sandén, 2016). The patients were between 20 and 70 years old, both women and men. The represented cancer illnesses were acute myeloid leukemia, head and neck cancer, esophagus cancer, prostate cancer and bladder cancer. The interviews were unstructured and lasted between two and three hours. The question was: “Please tell me about your lives.” Then the informants discussed various related topics while we as interviewers listened. In some interviews semi-structured questions, not pre-prepared, were used at the end of the interview to confirm previous analysis or to avoid misunderstandings. For example: “What did you mean when you said you didn’t believe them?” A total of 19 patients who had undergone different types of cancer treatments were included. All of the patients were considered cured or in disease remission. Semi-structured interviews with a specialized cancer care nurse and a cancer care physician were also conducted. The aim of these interviews was to explore seriously ill persons’ needs and concerns in everyday life and how they try to resolve them. The regional ethics committee at Lund University approved the study (Reg nr 2015:53).

### Theoretical sampling

In accordance with classic grounded theory, we theoretically sampled by making new decisions regarding what the next data collection would be after each interview (Glaser, 1978).

#### Part one: “Momentary contentment”

We started with interviewing elderly in groups of three with the one question “Please tell me about your lives”. This was a way to collect data from both what they said, how they said it and what they chose to/not to talk about. We then interviewed people in working ages to get age diversity of participants. When patterns and variations appeared in the analysis of data, we chose to collect new data as new questions arose. We continually interviewed the village’s oldest individual, 97 years of age, thus collecting notes from more than 10 hours from him talking about the village from a historical point of view. His stories were at the end of the study tested against data from the local historical and story-telling literature (Bottolfsen, 1995; Lauritzen, 2005; Rørtveit, 2008).

#### Part two: “Navigating a new life situation”

We conducted unstructured interviews in order to let the participating cancer patients decide what they wanted to talk about. Then we did two individual interviews to see if their content differed from focus group data. We also did two follow up semi-structured individual interviews to see if our interpretations from the group discussions differed from those particular participant’s views, which they did not. In these interviews we did not use a fixed form of questions, but notes from the previous group discussions (Sandén, 2016).

### Classic grounded theory analysis

#### Part one: “Momentary contentment theory”

New data was collected and analysed until further data did not provide any new information and saturation was reached. At this stage the formulated theory was modified in light of relevant existing literature (Glaser, 1998). Fieldnotes from both interviews and observations and then theoretical memos were written and drawn in various shapes and forms in the constant comparative analysis. Grounded theory focuses on incidents and memos rather than persons (Glaser & Strauss, 1967), and in this study the number of incidents coded and compared amounted to several hundred.

The memos were coded, categorized and sorted. After each interview or accrual observation new material was coded, analysed and compared with previous results until new data no longer gave new information, that is, saturation was reached (Glaser, 1998). The concepts were gradually developed to explain the informants’ attitudes toward life. The core category *Momentary contentment* was finalized in May 2014 and since then, memos and fieldnotes were written without discrimination, but interpretation and analysis was done selectively towards that core category. When no new information was reached through data collection all memos were compared to find relationships between categories and concepts letting theoretical codes emerge. Eventually a grounded theory of *Momentary contentment* was generated, explained by the sub-core categories: “Doing safety”, “Destiny readiness” and “Middle consciousness”.

#### Part two: “Navigating a new life situation”

The same classic grounded theory analytical method was used as described above. After each interview, new data was coded, analysed and compared with previous data until saturation was reached. What has emerged so far is not a saturated grounded theory but a conceptual description called “*Navigating a new life situation*”.

#### Part three: “Health navigation design”

*Navigating a new life situation* is compared to *Momentary contentment theory* and other research. Both memos and final analysis of the studies are compared in order to search for differences and similarities in needs and solutions, which is further discussed throughout this article.

### Fit, relevance, workability, modifiability and limitations

The results in a grounded theory study are not reports of facts, but rather probability statements about the relationship between concepts or an integrated set of conceptual hypotheses developed from empirical data. Grounded theory is thus judged by fit, relevance, workability and modifiability (Glaser, 1998, p. 18). Fit and relevance to both studies were achieved through continuous comparative analytical work. By focusing on what the informants chose as important topics we thereby allowed the main categories to emerge. The workability shows in momentary contentment theory how the core category, momentary contentment, frames our discoveries with the three main categories and explains what participants are doing to resolve their main concern. The cancer study has not lead to a new theory and has thus no workability of its own, but is used in the modifiability part. Modifiability of the studies was performed by connecting them.

Limitations are in the number of cancer patients involved and a lack of regional difference since they all come from a couple of regions in Sweden. The comparison between the two studies are, however, not based on regional area but in the context of life conditions. The regions of the cancer patients and the area where Momentary contentment theory has originally emerged are approximately 2000 kilometres apart. Momentary contentment theory has emerged from living in a context close to hazards and accidents, in so the context is similar to that of cancer patients. In order to fully know whether Momentary contentment theory has bearing on cancer patients on a more generalized level, we conclude that there is a possible fit and a need for more studies.

## Theoretical frame of references

Inspiration from Fraser (2005) allows us to further argue for redistribution of resources (e.g., new innovations) to enhance abilities for recognising specific needs among individuals providing opportunities for user representation in decisions. It would add to growing personal capabilities for participation, such as in promotion of personal emancipation and control as well as social inclusion. Inclusion of a patient voice is met by involving patients starting at the very beginning of the innovation design process. True patient inclusion in innovation requires “strong leadership to challenge traditional thinking and practices; a robust commitment to collaboration and partnership working; and a willingness to invest time in establishing a culture and infrastructure which values and promotes the patient perspective” (McNichol, 2012, p. 221).

In order to design a patient-centred psychosocial care we discuss innovation possibilities based in design thinking where each individual is the starting point and they themselves express needs and solutions. This is well in line with recent discussions on innovation within research in social work (Phillips & Shaw, 2011) and the emergent perspective raised by Essén and Lindblad (2013). In such a perspective we can gain deeper insights and better understanding of each patient’s context and needs, hence making a positive difference for those affected by the innovation. Christensen et al. (2009) argue that approximately 50% of consumed US healthcare is driven by physician and hospital supply, not by patients’ needs or demand. This clearly supports us in the endeavour to better understand and illuminate patient needs.

Existing innovation implementation ideas include frameworks such as Rogers’ (1983) innovation attributes for adoption, Glaser’s (2009) prescriptive factors and van Achterberg, Schoonhove and Grol’s (2008) evidence-based implementation strategies. Implementing innovation processes within social and health contexts has been difficult (May, 2013; Nilsson, 2014). Looking at design research the development within manufacturing industry has gone from product-orientation, via process-orientation, to cross disciplinary integration (Larsson, 2005). Lean thinking is a response to competitive pressure, whereas Agile thinking has to do with the sharing of resources, technologies and risks among companies (Larsson, 2005; Nagel & Dove, 1991). We argue similar needs for healthcare: a move from traditional healthcare to a cross-disciplinary care with a health focus design.

### Health promoting contexts

When looking for a holistic view on health, Aaron Antonovsky’s studies on health-promoting components became a natural source of data. Antonovsky developed the salutogenic theory. It connects cognition, behaviour and motivation and indicates sense of coherence (SOC) as the single most important ability to mentally survive hardship. A SOC consists of three components: comprehensibility, manageability and meaningfulness (Antonovsky, 1996); it is not bound by cultural context, but any culture can fit the concept in accordance with their culture (Antonovsky, 1996). The Momentary contentment theory emerged from applying classic grounded theory methodology to study the mechanisms behind dealing with life conditions before World War II in an isolated Arctic village where deaths, fishing boats perishing in ocean storms and tuberculosis and other diseases on shore were a natural part of life (Bottolfsen, 1995; Lauritzen, 2005; Sandén, Harrysson et al., 2015).

Both salutogenic theory and Momentary contentment theory have evolved from empirical studies of health in connection to various forms of hardship. But where Antonovsky chose to examine healthy-sick as opposing forces on a scale regarding what makes a person move towards health, our field studies have led us to focus on contentment, safety and an adaptive time perspective. An important base for momentary contentment is a surrounding frame of safety. Through altruism, cognitive tools, middle consciousness and an adaptive time perspective, a Sense of Safety is created in an otherwise unpredictable context. Sense of Safety is thus to *Momentary contentment theory* what SOC is to the salutogenic theory. We have neither in interviews nor observations in the momentary contentment study discovered the reflection of SOC that Antonovsky presents. None of the elderly in the Arctic expressed any meaning or SOC to their hardships–quite the opposite. Instead, they demonstrated a genuine ability to place uncontrollable difficulties in a *middle consciousness*, reformulating problems into solutions and using laughter as therapeutic confirmations. In other words, they were showing adequate ways of not having to reflect on purpose and meaning. With symbolic statements like “done with it” and “this is just the way it is” the informants moved on without requesting further context and meaning.

One main difference in the origins of salutogenic theory and Momentary contentment theory is the contexts in which they were studied. Antonovsky met concentration camp survivors and explored their needs and strengths (see, for example, Kvåle & Synnes, 2013). As for Momentary contentment theory, our informants lived in a partly isolated environment where accidents and deaths were still apparent. Where salutogenic theory and SOC evolved from people in need of coping with terrible things from the past, Momentary contentment theory emerged from an environment where people need to cope with what happens in the present moment. Momentary contentment shows how nature’s duality in the Arctic environment allowed hope and *destiny readiness* to live side by side in a collectivist spirit of enjoying life and feeling happy with what is; dealing with one moment after the other; placing the unmanageable in a *middle consciousness* creates a breathing space in the present–something often needed in a cancer context. Here and now the problem disappears, ready to be addressed when needed or when it becomes clear due to the need for help.

### Momentary contentment theory

Momentary contentment theory explains a culture of safety, joy and living in the moment. This is attained by activity, inclusion, altruistic helpfulness, acceptance of life changing events and an ability to separate negative from positive. Momentary contentment also separates the present moment from past and future expectations and has three categorical dimensions: (1) Creating safety, (2) Destiny readiness and (3) Middle consciousness.

#### Creating safety

Creating safety is a social norm that brings order through activity and contributes to actually being able to influence one’s own life, not only having a sense of doing so. Creating safety is based on a culture of helpfulness stemming from a time when helping out was a necessity for survival. Today it is rarely a question of life and death, but the altruistic helpfulness works contentment enhancing, both for those who help and for those who receive help. Creating safety is also visible through group responsibilities in including individuals and in collective concerns for shared functions, such as care and a local store (Sandén, Harrysson et al., 2015).

#### Destiny readiness

Destiny readiness is an individual mental positioning–a way of thinking where there are no expectations that life should be easy. Destiny readiness involves cognitive preparedness helped by linguistic tools for dealing with “what is”. An example is the expression “done with it”, which is used as a tool to move on from dwelling on something negative. Another expression “we know where we live” is used to accept the climates’ effects with, for example, recurring storms and flooding. Through linguistic expressions one can find ease in a rough situation (Sandén, Harrysson et al., 2015).

#### Middle consciousness

Middle consciousness is another mental positioning. It deals with worries that, despite the other Momentary contentment balancing mechanisms of Creating safety and Destiny readiness, are still present. Middle consciousness triggers a separation phenomenon; it gives an ability to switch between levels of consciousness and to place worries in a “standby” mode. In this standby mode, the healthy parts in people’s lives are highlighted without denying any illnesses or disabilities. Middle consciousness, together with Creating safety, includes offering help when help can be given, but otherwise promoting a focus on health (Sandén, Harrysson et al., 2015). This ability of separating parts of life brought on by Middle consciousness has its origins in a historically needed focus on the present moment, emphasizing the strength in every human being (Bottolfsen, 1995; Lauritzen, 2005; Rørtveit, 2008; Sandén, Harrysson et al., 2015). Momentary contentment is a theory with a potential for health promotion. When reviewing previous research, the psychosocial survival knowledge behind Momentary contentment shows a general but fragmented analogy with literature on what creates happiness, joy and satisfaction, linguistically as well as cognitively and behaviourally (Bauman, 2008; Egonsson, 2011; Haller & Hadler, 2006; Norman, 1998; Sandén, Harrysson et al., 2015). The theory is further explained in an article in *Grounded Theory Review* (Sandén, Harrysson et al., 2015).

*Creating the moment*–Past and future are relatively easy concepts to relate to, but what about the present moment? The perception of “now” can be seen as a momentary, barely observable tile in a chronological perspective; or as a phenomenological momentary situation, free from the environment in both time and space; or, like a subjective abundant experience, like a flow of the Greek kairos. Kairos means a time that does not follow the clock, and is perceived differently in different situations for different people. Kairos is a moment of indeterminate time in which an event of significance happens. Each now is a “critical juncture”, and every critical moment is a moment of kairos. That is because every moment creates the context in which the next moment will take place. Kairos is a golden opportunity to act in different ways to influence and change one’s fate, maybe just for the minute ahead or perhaps for life (Stern, 2004).

It is paramount in the Western world to establish, and relate to, a timely perspective. Studies on ex-prisoners living in dark rooms have demonstrated psychological problems and they often find a way to assess time. There is a need to find a coherent framework, regularity and predictability for the individual (Lasane & O’donnell, 2005). The social perception of time is central to a community’s culture and differences in the perception of time distinguish one culture from another. The view of time within a community is often hidden, unconscious and difficult for outsiders to perceive (Levine, 1996). Momentary contentment shows how time can be viewed with various references, such as seasons, and how an integration with the annual cycle helps create contentment when living in that environment. During the dark time people in the Arctic village sleep more, while summertime without sunset means reduced sleep, increased activity and even sleepless nights: allowing oneself to get tired of the darkness and then happily face the sun. This is done on a collective basis (Sandén, Harrysson et al., 2015). Translated into a patient perspective, one can learn to allow the body to become tired of treatments, but also joyfully meet a positive test result and create space for momentary contentment. This can be hard for relatives to recognize and explanatory models might be needed since the relatives are not living with the same time horizon.

### Momentary contentment and cancer patient needs

The results from interviews with cancer patients show similarities with Momentary contentment both in respect of experienced needs and perceived contexts. Both contexts have closeness to death and a need for help attached to them, and it is hard to predict who will be the next to get very ill or die. However, the cancer patient informants show different abilities in dealing with these needs. Our cancer patient study often showed a fragmented unknown life based on solitude instead of inclusion. *Creating safety* was seen in cancer patients’ yearnings for altruistic actions. Many cancer patients had found ways to use activity and helpfulness as tools for feeling better. However, they expressed difficulties in balancing their need for help with wishes to provide help for others. Inclusion was found within patient groups and organisations. However, they expressed difficulties in balancing their need for help with wishes to provide help for others. Inclusion was found within patient groups and organisations. However, *competitive parlance*, that is, comparing the seriousness and differences of treatments and prognosis and discussing them in a hierarchical way, was identified in the focus groups of patients sharing the same diagnosis. This verbal competition of cancer suffering worked contrary to inclusion. Yet, when mixing patients with different diagnoses within a focus group, competitive parlance was not seen. *Destiny readiness* was visible in our cancer patient study in the concept of hope where patients expressed uncertainties, misunderstandings and inconsistencies. All patients expressed a hope that “lives within”, where there is an opening for a *destiny readiness*, but many also expressed a feeling of being pushed by relatives, friends and healthcare staff towards being positive and cognitively expect hope. Thereby they also had thoughts about future fears and illness. *Middle consciousness* is apparent for cancer patients in their sporadic and fragmented attempts to navigate between feeling healthy and feeling sick with a fear of death.

## Results and discussion

*Momentary contentment theory* explains a culture where old survival strategies and patterns of behaviour live on. We have also chosen to listen to and to analyse cancer patients’ stories about their everyday lives rendering the concept *Navigating a new life situation*. Cancer patients expressed a need to navigate in a context where the illness creates a risky future as well as relational difficulties and shows a need for momentary coping strategies, for example to temporally encapsulate fear of death. In both the cancer patient navigating study and the Momentary contentment study the informants describe a life where death is a part of present life. They show similarities in needs, but where the cancer patients describe a new life situation with fragmented coping skills *Momentary contentment theory* shows how a well-developed collective external support system can create stability, a sense of belonging and security. This structure is something to fall back on when life is harsh (Sandén, Harrysson et al., 2015). Cancer patients expressed that healthcare staff continuity and knowing where to call to get help was very important to their psychosocial health. This is hard to achieve in todays’ fragmented healthcare system (Nilsson, 2014) and patients lack a *Sense of safety*. Holton (2007) explains in a grounded theory how too much change can lead to a loss of autonomy and identity for many knowledge workers. Holton suggests a need of re-humanization through fluctuating support networks: “*mutual engagement provides the arena for the release of collective creativity. It offers challenge, experimentation and learning. Mutual engagement builds confidence, commitment and energy. It enhances the bonding of network members*.” This connects to the Communities of practice, explained by Etienne Wenger (2000) as “groups of people informally bound together by shared expertise and passion for a joint enterprise–engineers engaged in deep-water drilling, for example, consultants who specialize in strategic marketing, or frontline managers in charge of a check processing at a large commercial bank”. Through virtual media, communities of practice could be created from a *health navigation design* perspective, improving patient skills and increasing knowledge in how to embrace momentary contentment.

### Applying destiny readiness and the complexity of hope

With a diagnosis that involves the risk of a shortened life, healthcare professionals frequently try to inspire hope. Attempts to inspire sometimes evolve into demands, thus cognitively tend to draw the patient from the present moment into thinking about an insecure future (Sandén, 2006). This is adjacent to Benzein’s conclusions about hope in palliative care, which distinguishes “living with hope” from “to hope for something”: “living with hope”, that is, being hopeful relating to what is present; “to hope for something”, that is, hoping relating to future and changes (Benzein, Norberg, & Saveman, 2001). Most of our respondents had a fairly good prognosis. However, they all shared thoughts and feelings concerning life and death. The interviews showed an importance of offering hope. But, in order for hope to be assimilated without evolving into a demand, it must comply with a person’s knowledge. Since our interviews were unstructured the patients decided what to share. They rarely mentioned the word hope but the phenomenon of hope was present in their stories. When talking they rather expressed concrete and symbolic ways to handle uncertainties. We found symbolism as well as concrete examples of actions, people or issues placed in between the present moment and possible death. It could be the surgeon symbolizing a personalized hope through magnificence “he will save me” or an activity plan of what to do between today and a possible relapse. A theory of equilibrium of hope shows how people create instinctive compensatory strategies to increase the existential hope, including the denial of life-shortening information or by increasing momentary enjoyments of life (Thulesius, Håkansson, & Petersson, 2003). One way to interact with patients on this subject, in line with *Momentary contentment* (Sandén, Harrysson et al., 2015), is to accept and respect the disease, patients’ knowledge and beliefs and to simultaneously accept that life with all its surprises, positive and negative, may go on. There is a need for more research on patients with possible deadly diseases, but where the present individual prognosis looks fairly good, and to position hope within a contented safety-enhancing context.

### Crisis management by middle consciousness and symbolic actions

A person’s subjective life situation experience is more important to subjective quality of life than the actual life situation. This refers to both medical and psychosocial factors, such as perceived health, close social relationships and perceived financial situation (Haller & Hadler, 2006). In other words, self-care and psychosocial innovations ought to be of great importance to healthcare improvements. By a *Middle consciousness* approach one can place illness in a standby mode and thereby separate sick from healthy; seeing disability when help is needed, and not seeing it when help is not needed. *Momentary contentment* indicates how it is possible to, at one point, act with helpfulness and then, at another point, treat the same person as fully fit, thus allowing all parts of a person affected by cancer to exist side by side (Sandén, Harrysson et al., 2015). In the cancer patient interviews we found small activities of health and normalcy to be symbolically helpful for patients in defining their healthy selves in relation to their illness and their symptoms. For example, while hospitalized they were doing something healthy like making a sandwich or changing clothes. This proved of great value in allowing the complete person to exist, the sick part alongside with the cancer part of oneself. Other examples were to allow fear to be expressed during a specified time frame or to keep a healthy part of social life alive while being hospitalized. These symbolical approaches were similar to *Momentary contentment* strategies but non-reflected and fragmentized. By bringing together *Momentary contentment* strategies with patient narratives we may be able to create new supportive tools; perhaps a pick and use manual in how to use different symbols, rituals and approaches in a personalized manner.

### Altruism induced contentment

In a summary of various research data on altruism a strong link is found between altruism and wellbeing, happiness, health and longevity–as long as helping others does not overwhelm a person. Altruism results in positive social inclusion, in distraction from personal problems and self-centred anxiety, in increased wellbeing combined with experiences of meaning and purpose and in a more active lifestyle. People who are involved in helping others generally describe their self-esteem as better than those not involved in such activities (Post, 2005). In a setting where helpfulness is based on altruism, each situation contributes to greater contentment for those involved. Altruism contributes to increased safety feelings for both those being helped and for those who do the helping. Each time altruism is practiced it increases a trust that you will not stand alone for future needs of help (Sandén, Harrysson et al., 2015). In our patient interviews this wish was expressed as a “need to be needed”. There is a need for a supportive psychosocial context. Support groups, for example, increase patient empowerment and lead to greater participation, increased search for knowledge about the disease and generally improve the patients’ abilities to navigate their disease (Weis, 2003).

### Inclusive contentment

A prerequisite for Sense of Safety–a feeling of enhanced safety in a community or group–is an ongoing inclusion process. Inclusion is established when individuals have a sense of belonging to a group and perceive themselves to be a distinct and unique member of it. This must be combined with a group responsibility of including the individual, rather than the individual connecting to the group (Jansen, Otten, van der Zee, & Jans, 2014; Shore et al., 2011). If the group does not take responsibility for such inclusion, it may lead to marginalisation of people who do not fall within the behavioural norms of the group (Ytterhus, 2012).

*Momentary contentment theory* explains how strategies for upholding heterogeneity strengthen inclusion processes within groups. Such strategies combine belonging with authenticity and include techniques for dealing with heterogeneity (Sandén, Harrysson et al., 2015). Many of these strategies can be modified to fit modern healthcare and be implemented within patient groups. By, for example, mixing different diagnoses in a group, many of the homogeneity problems of “not fitting in”, “being different” or “not being sick enough” that were apparent in our patient interviews are reduced.

### Humour and contentment

There are many testimonies of conversations between doctors and patients where serious health aspects are reflected in and by humour:

I think that what we regard as funny and what we regard as witty and sometimes sarcastic, are all part of what we regard as humorous. Whether or not something is humorous is whether or not it makes us giggle or smile or laugh. It’s interesting that there is a substantial literature about the health benefits of laughter. You sometimes hear the adage that laughter is the best medicine, so putting humour and laughter together is important. (Geriatrician Cornelius Foley, NYC, in interview about palliative care and humour, Monahan, 2015)

Humour is a *Momentary contentment* strategy containing both laughter and affirmation. Through humour a victimized person is drawn towards the present moment. This gives little room for dwelling and for expressions like “it will probably get better” or “time heals all wounds”, described by several cancer patients as provoking. Confirmation is rather created in laughter and contributes to contentment and an inter-subjective confirmation “I know you know I know” without a need to talk about what has happened (Sandén, Harrysson et al., 2015). During the patient focus groups, we observed similar confirmations where humour was used to move on and cope with situations.

### Nature brings contentment

Sceneries bring another kind of support. *Momentary contentment theory* shows how people’s relationship to nature, although different, bring similar internal power enhancement (Sandén, Harrysson et al., 2015). The role of nature did not come up as an issue in our study of cancer patients, but there are several studies that show nature’s role as a healing force (Sandén, Harrysson et al., 2015; Strang, 2007). Aside from specialized green rehabilitation stations, hospitals very often miss the point of nature’s role in people’s minds. Technology exists, both interactive and non-interactive, that can create environments that partially fill a similar function, for example moving panoramas. By an innovative linking of technical, medical and psychosocial knowledge we have a unique multi-disciplinary potential to contribute to better psychosocial health among patients and families.

### Bridging knowledge

Above are areas of possible innovative fits between *Momentary contentment strategies* and cancer patients’ testimonies on a general level. In order to make them become non-fragmented innovation processes these areas need to be linked together in a personalized manner for individual patients. Already existing psycho-pedagogical tools to increase knowledge about the disease show that specific and clear disease information increases patient empowerment and participation. From participation grows knowledge (Alden, 2014; Kane, 2014; Schmidt et al., 2015). There are several studies as well as patient testimonies pointing to the importance of patient participation and self-action in both diagnostic and treatment processes (deBronkart, 2011; McDonald, Bryce, & Graber, 2013). However, we argue, there is a need to design solutions as a whole and not look at each issue separately.

## Future research

The presented areas, aligned in comprehensive programmes allowing patients to be active as well as interactive, are potential innovations in today’s fragmented healthcare. We suggest that the modification of *Momentary contentment theory* to fit with cancer patients *Navigating a new life situation* outlined in this paper is a starting point of an explorative implementation route for the presented areas into a healthcare context. There is certainly a need to further investigate active patient centred health promoting processes and structures in the continuum of care. Nonetheless, as problematized in May’s (2013) studies of innovation processes within social and health contexts, resulting in the Normalization Process Theory (NPT), there are a number of difficulties when it comes to promoting change in health care contexts. The NPT is suggested to provide understanding of the actual work that goes on in the socio-political context as well as the socio-technical practices. There are both structural and process-related difficulties obstructing implementation to be addressed in general as well as in specific situations (May, 2013; Nilsson, 2014). Furthermore, as concluded by Page (2014, p. 230) “the best way to innovate is from getting people together with different skill sets and devising questions that need to be answered.” Hence, it includes patients, relatives, staff, politicians and researchers as well as future, yet unknown actors. By combining *Momentary contentment theory* (Sandén, Harrysson et al., 2015) and Patient Process Orientation (Nilsson, 2014) with NPT (May, 2013) and a health navigation design perspective we suggest that contentment enhancing innovation practices such as virtual communities of practice (Wenger, 2000) or fluctuating support networks (Holton, 2007) is an approach well worth trying.

## Conclusion

By analysing interview and observational data using grounded theory we found similar everyday challenges between people in Arctic Norway and Swedish cancer patients. In Arctic Norway, *Momentary contentment theory* is the grounded theory conceptualization that explains how creating a Sense of Safety and room for activity and joy in the present yields well-being. We suggest that implementing *Momentary contentment* approaches for cancer patients would help them to improve *Navigating a new life situation*. This would be achieved through an adaptive view on time in combination with cognitive tools helping patients to act on what is possible to influence and to simultaneously let go of that which is not. Thus, “space-of-moments” with a *Sense of Safety* can be created in an unpredictable context. Patients’ tactics could change from today’s fragmentized attempts to the concrete and explainable by a user’s manual of *Momentary contentment* strategies that also would make it easier for patients, family and friends to understand the patient’s situation and actions. Finally, we suggest that through a health navigation design, the contentment safety-enhancing mechanisms from *Momentary contentment theory* may contribute to capacity building and eventually enhance quality of life.
